# Human Factors and Airway Management in COVID-19 Patients: The Perfect Storm?

**DOI:** 10.3390/jcm11154271

**Published:** 2022-07-22

**Authors:** Gerardo Cortese, Massimiliano Sorbello, Ida Di Giacinto, Martina Cedrone, Felipe Urdaneta, Luca Brazzi

**Affiliations:** 1 Department of Anaesthesia, Intensive Care and Emergency, AOU Città della Salute e della Scienza, Corso Dogliotti 14, 10126 Turin, Italy; cortesegerardo@gmail.com (G.C.); luca.brazzi@unito.it (L.B.); 2 Anesthesia and Intensive Care, AOU Policlinico San Marco, 95121 Catania, Italy; 3 Anesthesia and Intensive Care, Mazzoni Hospital, 63100 Ascoli Piceno, Italy; digiacintoida@gmail.com; 4 Department of Surgical Sciences, University of Turin, 10126 Turin, Italy; marticed93@gmail.com; 5 Department of Anesthesiology, North Florida/South Georgia Veteran Health Systems, University of Florida, Gainesville, FL 32608, USA; furdaneta@anest.ufl.edu

**Keywords:** COVID-19, critical care medicine, airway management

## Abstract

The SARS-CoV-2 pandemic heavily impacted healthcare workers, increasing their physical and psychological workload. Specifically, COVID-19 patients’ airway management is definitely a challenging task regarding both severe and acute respiratory failure and the risk of contagion while performing aerosol-generating procedures. The category of anesthesiologists and intensivists, the main actors of airway management, showed a poor psychological well-being and a high stress and burnout risk. Identifying and better defining the specific main SARS-CoV-2-related stressors can help them deal with and effectively plan a strategy to manage these patients in a more confident and safer way. In this review, we therefore try to analyze the relevance of human factors and non-technical skills when approaching COVID-19 patients. Lessons from the past, such as National Audit Project 4 recommendations, have taught us that safe airway management should be based on preoperative assessment, the planning of an adequate strategy, the optimization of setting and resources and the rigorous evaluation of the scenario. Despite, or thanks to, the critical issues and difficulties, the “take home lesson” that we can translate from SARS-CoV-2 to every airway management is that there can be no more room for improvisation and that creating teamwork must become a priority.

## 1. The SARS-CoV-2 Pandemic

The severe acute respiratory syndrome novel coronavirus 2 (SARS-CoV-2) is responsible for a multifaceted disease named the coronavirus disease 19 (COVID-19), which spread around the world, cumulatively causing over 514 million confirmed cases and over 6 million deaths globally since the beginning of the pandemic (WHO data) [1].

The consequences of such a pandemic posed tremendous challenges to the whole world, with influence on social life, work, trading, and travelling [2], and last but not least, it resulted in tremendously increased pressure on every healthcare system, down to each individual hospital in charge of COVID-19 patients, and particularly on Emergency and Intensive Care departments, with an enormous workload for healthcare workers (HCWs), often operating in sub-optimal if not adverse conditions.

The physiopathology of the new disease is still under research, with many questions still unanswered [3]. Vaccination programs have started in many countries all around the world, but the global challenge is far from finished.

One of the initially underestimated aspects of the pandemic, especially in the first months of spreading, was the psychological impact of so different working environment and conditions on HCWs, who were constantly bombarded by a series of previously unknown stressors [4].

## 2. SARS-CoV-2-Related Stressors

In the attempt to provide a schematic representation, we have divided the COVID-19-associated stressors into those regarding the patient and those affecting HCWs.

Sick patients obviously experience the psychological impact of the disease, including the fear of death, loneliness and distance from families and friends, including the *stigmatization* phenomenon: they may feel themselves to be different or dangerous to other people, including the HCWs who are taking care of them. They may extend this feeling also far beyond the acute phase of sickness while still testing positive, developing avoidance, escaping and isolation behaviors.

HCWs are differently affected: as scientists, COVID-19 may represent, or may have represented, a challenge for clinical and bench research, igniting enthusiasm and the sense of challenge [5]. The still uncomplete understanding of this “new” disease and the need to change the decision-making process yet starting from the triage has led, in over 60% of cases, to tension at work or to the inability to carry out daily activities once back home [6]. Moreover, the daily impact of dealing with poorly effective therapies, with death and with impotence, tiredness and repetitiveness may easily disrupt the “positive” feeling, changing it into a sense of failure which amplifies the stress itself.

The fear of becoming infected or of infecting family and friends may trigger anxious or depressive states, leading to a tendency to self-isolate and a reduction in empathy and emotivism, once again fueling the stress chain [7]. Empathy with patients plays also a pivotal role: as anesthesiologists and intensivists, we are often called to support vital functions of unconscious patients, which do often represent “a case” over an individual. From a certain perspective, this is also a defense mechanism to avoid adsorbing the negativity deriving from repeated negative and stressing situations. The COVID-19 patient is often different, given that we often meet these patients during the “happy hypoxia” [8] phase, when they are fully conscious, fighting the disease and at same time its fear. We unavoidably develop empathy with some patients more than with others, we know their names and stories, and we are often called to accompany them along their worsening pathway of oxygenation, explaining to them the need for intubation and often representing the last person they talk to before an unnatural sleep which may lead them directly to death. The worst feeling remains the awareness of being at high risk of becoming victims to the same condition the patients are suffering from [9].

Overwork, team-related factors (lack of experience and sense of inadequacy, unfamiliarity with new colleagues), organizational factors (lack of resources or personal protective equipment, missing protocols and operative instruction) fatigue and the dull and repetitive pattern of identical shifts do ultimately contribute to create the perfect storm of burnout and stress [10,11,12].

Anesthesiologists and intensivists are actually considered amongst the categories with the poorest psychological well-being and with the highest stress and burnout risk [13], the literature and data highlighting an increase of about 50% of new cases of anxiety and burnout and up to 30% of symptoms related to depressive patterns [14], including post-traumatic stress disorders and suicidal attempts. Data from the Wuhan area indicate that front-line HCWs, especially women (76.7%) and nurses (60.8%), developed symptoms of depression (50.4%), anxiety (44.6%), insomnia (34.0%) and mental distress (71.5%) [15].

Regarding anesthesiologists and intensivists, do we have particularly high-stress situations? The answer is probably yes, and airway management is definetely one of them.

## 3. Airway Management in COVID-19 Patients: A Stress within Stress?

The airways of COVID-19 symptomatic patients may exhibit viral loads up to 60 times of non-symptomatic patients [16], with a direct correlation shown between viral load and the risk of intubation and mortality [17]. Recent studies highlight that intubation and extubation are high risk aerosol-generating procedures [18] and that HCWs exposed to such procedures have a higher risk of contamination and infection [19,20,21], which has been recently estimated as over 1 in 10 for HCWs performing airway management [22].

In the specific setting of COVID-19 airway management, apart from infective risks, we may list a long series of pitfalls and difficulties.

Much has been written on algorithms and recommendations [8,23,24,25,26,27] to guide operators on technical and clinical paths when facing a COVID-19 patient. Nevertheless, debate still remain [28,29,30] and the implementation and knowledge of guidelines is not capillary [31], with differences between different settings and ICU [32,33].

In any case, some situations may not even be changed despite available guidelines: the atypical hypoxemic patterns of COVID-19 patients may lead to postpone intubation and then to face severely hypoxemic patients with compromised respiratory function and a very short apnea time [34], not without emotional implications and psychological pressure.

The lessons from the National Audit Project (NAP) 4 [35] taught us that safe airway management is based on the four cornerstones of mindful preoperative assessment, the planning of an adequate strategy, the optimization of setting and resources and the rigorous evaluation of the airway scenario. Real life application of any of them will not be that easy if there is no type of pre-procedural strategy planning and if we deal with COVID-19 patients the same way we treat non-COVID patients.

Logistic issues also complicate routine management. The use of PPE is responsible for a series of issues and limitations: decreased movement and reduced tactile sensitivity because of multiple gloves result in the loss of dexterity and precision [36], with some procedures lasting longer [37] and becoming more complicated [38,39], with a serious risk to increased airway trauma on COVID-19-inflamed airways, especially when using devices such as tracheal introducers [8,40,41]. All these effects may be even amplified if HCWs are worried about the lack of PPE or improper donning procedures, with consequential unnatural movements and the avoidance of behaviors considered to be higher risk [42]. The use of barrier enclosures in place or together with conventional PPE may also not only increase risk of infection but also hinder airway management [43], triggering emotional responses and cognitive biases. Goggles or face shields may become fogged or tarnish with prolonged use, thus limiting visual acuity. Prolonged shifts while wearing PPE may cause discomfort, limitations, overheating, sweating and hypercarbia, with implications on mental stress and anxiety [44], which may also result in memory and attention reduction and impaired performance, increasing the risk of troubles during airway management and of self-infection during donning or doffing PPE, and last but not least, PPE complicates the identification of team-mates and interactive communication during procedures [8]. Running for an intubation in a ward or emergency department may also turn into a challenge, because of an unfamiliar environment, unknown team-mates, lack of equipment and spatial limitations [33].

Despite being recognized by the recent literature as main responsible of airway accidents [45,46,47,48,49,50], less attention has been focused on non-technical issues with COVID-19 airway management.

Tiredness, the fear of infecting or becoming infected and the lack of familiarity with colleagues, teams and locations may all result in anxiety and performance deterioration. Difficult communication, patient empathy, psychological pressure coming from rapidly deteriorating vital parameters and many other factors do contribute to inducing anxiety and the consequent tendency towards cognitive biases and to non-technical errors, with unavoidable consequences on performance and success.

It would then be a judgement error not to consider airway management as a powerful stressor against the stressful background of the COVID-19 pandemic.

This consideration has a bi-directional implication: if, on one hand, airway management increases stress, on the other hand, this stress itself may hinder airway management, generating a vicious circle whose center is represented by the human factor [51].

The unrecognized or underestimated recognition of both physical and mental well-being failure may result in dexterity deterioration and poor teamwork, loss of resilience [52], with negative effects on patient safety and quality of care [53]. The need for a careful monitoring of the psychological condition of operators in order to manage long-term psychological outcomes and alleviate the psychological burden of the pandemic on frontline staff then becomes a priority of paramount importance [54].

Is it therefore necessary to transform the multi-complexity of these patients into a positive challenge to improve the outcome of these patients? Is it necessary to review the airway management strategy of these patients?

## 4. The Magic Bullet for Success in One Word: Teamwork

Sir Charles Darwin stated that “It’s not the strongest species, but the promptest to change that survive”, so it is likely that our daily ability to readjust to new clinical and organizational needs is the key to success in COVID-19 airway management, not forgetting the adoption of psychological support tools for HCWs.

Considering any COVID-19 intubation as a difficult one may be a positive trigger: physiology, logistics, mental stress, all support this statement, and dealing with any intubation, as with a difficult one, may induce preparedness and pre-emptive corrective behaviors. Lack of planning leads to failure, especially when the airways are difficult, thus the assumption of an “anycase” difficult airway would be a powerful trigger for developing a strategy and to share and discuss it within the team.

Anticipating intubation in rapidly deteriorating patient may also be a parallel approach; this does not mean an aggressive intubation approach, but a precise identification of evolutive cases (for example adopting modified early warning scores or prognostic scales [55]), so to arrange intubation before the patient’s conditions become critical.

Based on this approach, planning should include a preparatory phase, with a full briefing to be performed before entering the intubation room, with the identification of staff members and respective roles; the preparation of a dedicated intubation cart with available algorithms and cognitive aids in the room; the definition of a precise plan, including backup and rescue; and the arrangement of precise gestures and strategies to improve communication [8,23]. Any instructions during airway management should be simple, and expressed clearly and loudly, keeping the communication flow bi-directional through feedback. Especially in case of new team-mates, the simple placement of a sticker with the name of the individual on the top of the visor or on the hazmat suit may help and favor communication [35]. The pre-procedural identification of roles may also include localization of the team members in the room, which will enhance and facilitate roles distribution (who helps with airways, who delivers medications and checks the monitor) and should thus be considered before initiating any procedure [8]. Such an approach may heavily contribute to visualizing each procedural step in advance, to compensate the physical restrictions imposed by PPE and to reduce physical and cognitive workload.

The choice of equipment is essential. With a wide range of airway management devices currently available, it is advisable to limit the choice to a few, as too wide a range compromises speed and efficiency when needed. Strong standardization, ideally shared within the team, is highly recommended, so that every member of the team, as they may change over time, knows exactly which device is available, how to use it, where to find it and how to keep it functional. Knowing the environment and having certainties is the key to avoiding uncertainty, especially when working in stressful and unfamiliar contexts [33,56].

“Testing” new techniques or using them for the first time or with a lack of specific training in the critical setting of a COVID-19 intubation may be deleterious and should carefully be avoided [23,57]. This also applies to the video laryngoscope, the first-choice device for the intubation of COVID-19 patients [8,23,58], the use of which should be adopted only if adequate training has been previously given.

In such a perspective, despite desperate times, we should not adopt desperate measures [59], but only rely on certified devices, including PPE [43], tested devices and techniques, abandoning the MacGyver bias in favor of scientific evidence [60].

The adoption of checklists and cognitive aids reduces workload, mental stress and improves performance [61]. This is even more true with COVID-19 intubations, and many cognitive aids and approaches have been proposed [62,63] as a simple but powerful decisional support tools to maintain a high level of concentration and preparedness thorough all airway management procedure, including extubation.

Extubation can be as risky as intubation [35], and in terms of aerosol generation it is probably even more dangerous than intubation or any other airway maneuvers [18]. It is therefore necessary to maintain a high level of attention, to try to identify the patient at greatest risk when planning extubation [64], adopting the same non-technical principles of intubation.

The magic recipe to achieve these goals is establishing teamwork. The specificity of these patients makes the creation of dedicated high skilled anesthesia based teams responsible for airway management strongly advisable, possibly following a shared protocolized tracheal intubation model [65,66], and ideally centralizing intubation spots in familiar and well-organized environments [33]. A team means sharing decisions and responsibilities, it means adding value which is more than the sum of its parts (i.e., the individual values of the team members), amplifying the performance and diminishing risks through mutual control and interaction.

As a further point, the team also allows the creation of an emotional platform where positive and negative feelings and perceptions may be, respectively, amplified, through the sharing of success, or mitigated, through the distribution of responsibilities, workloads and mutual support.

In this perspective, we may say that COVID-19 created a distance paradox: in a world where social distancing is a key policy to containing viral spread and diffusion, the virus created the premise of bringing together people through non-physical means, creating strong working groups, worldwide research groups without boundaries of ethnicity and nationality and a transversal empathy and sense of community.

The word “preparedness”, literally the state of being prepared for a particular situation [67], sums up well the goal we must strive for with our team [56]. In the context of the Department of Anesthesia, this requires different levels of intervention: at the team level, it will be necessary to focus more on human factors, in order to increase the safety of the whole group, while individually it will be necessary to work on personal skills in order to increase the efficiency of each HCW.

Preparedness also means optimizing equipment and materials by verifying that all that is needed is available and that all operators understand both the devices and how to use them. The transition from simple competence (“I know how to do something”) to experience (“I’m confident in doing it correctly and in transferring my knowledge”) is a crucial point and requires a coded and regularly applied training program.

After each airway management procedure, it would be also advisable to carry out a debriefing of the team, a sort of critical review of what happened. This should be short and structured, following the steps of TALK (Target, Analysis, Learning Points, Key Action) [68], remaining open to any input so as to allow the continuous search for solutions or improvements.

Last but not least, the power of simulation may also help in this insidious setting, as clearly demonstrated by many studies also performed in the COVID-19 context, for either technical (PPE [69], airway devices [70]) and non-technical scenarios. Interestingly, by enhancing technical skills and awareness of non-technical pitfalls, simulation also turns out to be a powerful tool to reduce anxiety and to develop resilience [71], once again highlighting its important role in the modern training and teaching of airway management.

## 5. The Future

The future of the COVID-19 pandemic depends on many factors, including whether people will develop lasting immunity to the virus, including the efficacy of vaccination programs, whether seasonality will affect its spread, and—perhaps most importantly—the choices that governments and individuals will adopt [72].

SARS-CoV-2 will not end soon, and it has dramatically changed social life and healthcare systems; the future will unavoidably be hybrid, with a scenario of forced coexistence with this invisible enemy. The healthcare systems of the near future will necessarily develop hybrid and flexible programs to adapt to the waves of epidemic and endemic phases of infections, allowing on one hand the safe and effective care of COVID-19 patients but on the other the maintenance of surgical and medical activity for non-COVID patients.

In this perspective, although symptomatic patients are more likely to transmit the virus, asymptomatic patients can also be infectious [73], thus it is essential to manage all airway procedures as high-risk [65], and we will probably also need to implement different approaches to airway management in our routine practices. Technical skills will maintain the same importance, but non-technical issues need to be empowered, taught and developed, also through simulation teaching programs. The human factor perspective needs to be further implemented and recognized as cornerstone in the proficient and safe management of any airway.

Given the psychological issues associated with the pandemic, there is a strong need at any level of developing and promoting mental well-being policies: HCW need to cope with their daily activities and find new balances through resilience and team sharing. Institutions need to recognize the risks and pitfalls associated with HCWs’ physical and mental overload, promoting health, social and support policies. This may include the adoption of nationwide telematic psychological assistance counseling services or promoting, as with the military or police officers, programs such as Trauma Risk Management (TRIM) training [74] and any other valuable policy to mitigate the multifaceted impact of the pandemic [75].

COVID-19 is a challenge we will not win as individuals but only through the development of teamwork and team-thinking. To face an unprecedented perfect storm, we need to switch to unprecedented approaches, so to effectively respond to the need for adaptation and changes, as foreseen by Sir Charles Darwin.
